# Iberian red deer: paraphyletic nature at mtDNA but nuclear markers support its genetic identity

**DOI:** 10.1002/ece3.1836

**Published:** 2016-01-28

**Authors:** Juan Carranza, María Salinas, Damián de Andrés, Javier Pérez‐González

**Affiliations:** ^1^ Ungulate Research UnitCátedra de Recursos Cinegéticos y Piscícolas (CRCP)Universidad de Córdoba14071CórdobaSpain; ^2^ Instituto de AgrobiotecnologíaCSIC‐UPNA‐Gobierno de Navarra31192MutilvaNavarraSpain

**Keywords:** *Cervus elaphus hispanicus*, conservation genetics, Iberian glacial refugia, paraphyletic taxa, phylogeny, phylogeography

## Abstract

Red deer populations in the Iberian glacial refugium were the main source for postglacial recolonization and subspecific radiation in north‐western Europe. However, the phylogenetic history of Iberian red deer *(Cervus elaphus hispanicus)* and its relationships with northern European populations remain uncertain. Here, we study DNA sequences at the mitochondrial control region along with STR markers for over 680 specimens from all the main red deer populations in Spain and other west European areas. Our results from mitochondrial and genomic DNA show contrasting patterns, likely related to the nature of these types of DNA markers and their specific processes of change over time. The results, taken together, bring support to two distinct, cryptic maternal lineages for Iberian red deer that predated the last glacial maximum and that have maintained geographically well differentiated until present. Haplotype relationships show that only one of them contributed to the northern postglacial recolonization. However, allele frequencies of nuclear markers evidenced one main differentiation between Iberian and northern European subspecies although also supported the structure of both matrilines within Iberia. Thus, our findings reveal a paraphyletic nature for Iberian red deer but also its genetic identity and differentiation with respect to northern subspecies. Finally, we suggest that maintaining the singularity of Iberian red deer requires preventing not only restocking practices with red deer specimens belonging to other European populations but also translocations between both Iberian lineages.

## Introduction

During the Quaternary ice ages, most of northern Europe was covered by ice, while permafrost extended through Central Europe, shaping the distribution of many temperate species to ice‐free glacial refugia during the Late Pleistocene (Hewitt 2000). According to Sommer and Zachos (2009), during the last glacial maximum (LGM, 25,000–18,000 BP), the distribution ranges of extant species were more contracted than ever before in their history. Main European glacial refugia corresponded to the southern peninsulas of Iberia, Italy, and the Balkans. These three main refuges acted as sources for the postglacial recolonization of the continent for different species, carrying distinct genetic lineages as a legacy of their long isolation from each other (Taberlet et al. 1998; Hewitt 1999; Torroni et al. 2001; Schmitt 2007; Knopp and Merilä 2009).

Various phylogeographic analyses have pointed to the Iberian Peninsula as the main refugium for postglacial recolonization of north‐western Europe for different animal species (e.g., insects, Vila et al. 2005; amphibians, Martínez‐Solano et al. 2006; reptiles, Paulo et al. 2008; Miraldo et al. 2011; mammals, Branco et al. 2002; Vernesi et al. 2002; Centeno‐Cuadros et al. 2009). Moreover, the Iberian Peninsula harbors a high physiographic complexity that, in combination with concurrent Mediterranean and Atlantic influences responsible for a wide range of climates, may favor the presence of subrefugia (Gómez and Lunt 2007) and opportunities for intraspecific divergence (rev. Schmitt 2007).

Phylogeography is normally constructed from intraspecifically variable markers with phylogenetic signal such as DNA sequences (Lowe et al. 2004). Specifically, mitochondrial DNA has proven to be an ideal sequence for phylogeographic analyses due to its high rate of sequence evolution, uniparental inheritance, and lack of recombination (Avise 2000; Lowe et al. 2004). Other markers such as allozymes, RAPDs, or microsatellites provide information about absolute differences but no information about genealogical relationships (e.g., Harris 1999). In particular, microsatellite markers have shown to bear high rates of homoplasy (same alleles but different ancestors) that might hinder the correct establishment of phylogenetic relationships between species (Estoup et al. 2002). However, microsatellites can be useful in solving particular questions that emerge in phylogeographic studies, mainly those affecting the divergence of closely related taxa over time periods shorter than those normally required to find signatures in mtDNA (Estoup et al. 2002). Postglacial recolonization of northern Europe after the last glacial maximum (LGM) from southern refugia may have taken place as recently as 16,000 years BP (Meiri et al. 2013), which is a short time to be tracked by mtDNA alone. By contrast, microsatellite markers can provide information for the most recent events during postglacial recolonization (e.g., Hewitt 1999; Palo et al. 2004; Knopp and Merilä 2009; Dool et al. 2013). Furthermore, information provided by microsatellite markers is especially important in species in which males are the dispersing sex and females are philopatric (Petit and Excoffier 2009). This is because the existence of taxonomic distinctiveness found at mtDNA can be prevented by male‐driven gene flow (e.g., Nater et al. 2011).

The red deer *(Cervus elaphus)* is the most widely distributed species of large wild mammal in the main the Palearctic (Geist 1998). It is an interesting model species whose distribution and genetic structure have been shaped by both natural and anthropogenic factors (Skog et al. 2009), with many recent human‐mediated demographic and biogeographic alterations that might blur the basal structure shaped by natural selection and biogeographic phenomena during the last ice age (Zachos and Hartl 2011). Paleontological findings have identified the Iberian Peninsula along with southern France, and southeastern Europe from the Balkans up to the Carpathians, as refugial areas for the red deer during the LGM (Sommer et al. 2008). Studies on red deer phylogeography in Europe based on mitochondrial DNA (Ludt et al. 2004; Skog et al. 2009; Niedziałkowska et al. 2011) found three main lineages: a western lineage (A) that spread from Iberia through the British Isles to Scandinavia, Germany, and western Poland; an eastern lineage (C) that originated in the Balkans, the Romanian Carpathians and expanded northwards to Austria, Hungary, and the Czech Republic; and a third lineage (B) confined to Sardinia and northern Africa (Ludt et al. 2004; Skog et al. 2009). Recent analyses by using both modern and ancient mitochondrial DNA have confirmed that areas in north‐west Europe were recolonized by red deer from the Iberian Peninsula after the last glacial maximum (Meiri et al. 2013).

Since red deer populations persisted throughout the entire last glacial period in the Iberian Peninsula (Sommer et al. 2008; Meiri et al. 2013), the identification of refugial genetic clades and their current distribution may provide important insights into the historical processes underlying the patterns of intraspecific variation in Iberian and other west European lineages. The most comprehensive analysis so far for Iberian red deer *(C. e. hispanicus)* phylogeography was recently performed by Fernández‐García et al. (2014). In that study the authors suggested the existence of two haplogroups related to two glacial refuges for red deer within Iberia. However, their phylogenetic differentiation remains uncertain from those results (see Carranza and Martínez 2014). Additionally, information on the degree of genetic differentiation at nuclear markers between the Iberian and with other west European lineages is lacking, which is especially important because such differentiation presumably took place very recently during postglacial recolonization.

Here, we use mtDNA and nuclear genetic markers to study the phylogeography of red deer. We focus on the study of current Iberian red deer lineages in the context of West European red deer, to address the following objectives:

1. To study the haplotype composition and phylogenetic relationships between Iberian and other European clades.
2. To infer the history of phylogenetic differentiation between Iberian clades.
3. To assess the genetic structure of red deer in western Europe using microsatellite markers.
4. To provide basis for decisions on preserving the current main Iberian populations from foreign restocking

## Material and Methods

### Study area and sample collection

Most of current distribution of red deer in the Iberian Peninsula has resulted from many translocations, some of them documented, and others probably not, from a few remaining populations (Cabrera 1914; Blanco 1998; Carranza 2010). We do not have evidence of any current red deer population in Spain that have been established mainly by restocking with non‐Iberian red deer, although some individuals or stocks exist at particular hunting estates that may belong to other European subspecies or may be hybrids between Iberian and foreign red deer (Carranza et al. 2003; Carranza 2010; Fernández‐García et al. 2014). According to the available information (Soriguer et al. 1994; Crespo 2013), we can consider Extremadura, Sierra Morena, and Montes de Toledo as the main areas where native populations persisted, roughly throughout a large discontinuous region in western and central‐southern Spain. Most current populations in the north of Spain, Portugal, and some southernmost ranges have resulted from translocations from these native remaining stocks. Therefore, we focused our sampling effort mainly on central‐south and western Spain, with some samples from northern and southern populations. Also, we included some samples from other European populations to study their relationships with Spanish populations.

We obtained samples of tissue (ear or muscle pieces) or antlers (antler‐bone shavings) from 611 individuals from wild populations of red deer in Spain (Iberian Peninsula) (Table 1; see below Fig. 3 for the geographical location of sampling sites in relation with red deer distribution in Spain). Samples from other countries included Norway, Scotland, Germany, and Italy, making a total of 682 sampled individuals (Table 1).

**Table 1:** Red deer samples used in the study. Table shows nominal subspecies, sampling sites, number of samples (*N*) used for mtDNA and nuclear DNA analyses, and location in map of Figure 3 for Spanish samples. Asterisks denote Spanish populations that most likely correspond to native remaining stocks (based on Pérez et al. 1998; Carranza 2011, and J. Carranza unpublished information). Subspecific names were assigned on the basis of location of samples plus additional information if available. For Italy, red deer was reintroduced in this country from eastern Europe, hence *C. e. hippelaphus*

Subspecies	Sampling sites	*N* (mt DNA)	*N* (nuclear DNA)	Map location
*Cervus elaphus hispanicus*	Alcornocales	7		1
	Barcelona	48		2
	Burgos	2		3
	Cardeña*	138	20	4
	Despeñaperros*	5		5
	Doñana*	1		6
	Hornachuelos*	203	19	7
	Madrid*	31		8
	Monfragüe*	15		9
	Montes de Toledo*	20	20	10
	Montes Universales	2		11
	Navarra	4		12
	Sierra de Baza	2		13
	Sierra de la Culebra	2		14
	Sierra de San Pedro*	86	19	15
	Sierra Madrona*	4		16
	Sierra Norte de Sevilla*	9		17
	Valle de Arán	10		18
	Zaragoza	22		19
	**Spain (Total)**	611	78	
*C. e. atlanticus*	Norway	21	20	
*C. e. scoticus*	Scotland	20	20	
*C. e. elaphus*	Germany	22	20	
*C. e. hippelaphus*	Czech Republic	1		
	Italy	7		
	**Total Samples**	682	138	

For microsatellite analyses, we selected balanced subsamples in those populations located in the main native areas and for which we collected at least 20 samples (see Table 1 and Fig. 3 below for location). Contrary to mtDNA sequences, allele frequencies at microsatellite loci are highly sensitive to alterations in the composition of populations. Newly created populations at hunting areas might present artificially altered genetic compositions caused by founder effects or bottlenecks (see Haanes et al. 2011). To avoid this problem, we decide to select only the main native areas for the study with nuclear markers and to use balanced number of samples from them to allow genetic diversity comparison between areas. Therefore, we selected samples from Sierra de San Pedro (west Spain, location 15 in Fig. 3; *N* = 19), Cardeña (south Spain, location 4 in Fig. 3; *N* = 20), Hornachuelos (south Spain, location 7 in Fig. 3; *N* = 19), and Montes de Toledo (central Spain, location 10 in Fig. 3; *N* = 20). Additionally, we selected 20 samples from areas in which the western European lineages were described (Ludt et al. 2004; Skog et al. 2009; Niedziałkowska et al. 2011): Norway, Scotland, and Germany.

Antler shavings were preserved dry at room temperature and tissues at −20°C in absolute ethanol.

### Laboratory procedures

DNA extraction was performed by a standard salting‐out procedure (Miller et al. 1988). Extracted DNA was loaded in agarose gel in order to assess the integrity and quantity of material. For mtDNA sequences, polymerase chain reaction (PCR) amplification of 922 bp of the mitochondrial control region (mt CR) was performed using primers pairs PRO/PHE (Wood and Phua 1996) in a 20 *μ*L reaction mixture containing: 1× PCR buffer, 0.2 mmol/L dNTPs, 0.1 *μ*mol/L of each primer, 1 mmol/L MgCl_2_, 1 U of Taq DNA polymerase (Bioline, London, UK), and 50–100 ng of template DNA. The thermal cycling profile was 94°C (3 min) followed by 35 cycles at 94°C (1 min), 56°C (1 min), and 72°C (1 min) with a final extension step of 72°C (1 min).

For sequencing, 10 *μ*L of the PCR product was purified with 0.2 U of shrimp alkaline phosphatase and 0.4 U of Exonuclease I (FERMENTAS, Burlington, Canada) with an incubation of 37°C for 30 min followed by 85°C for 15 min. We sequenced the mt CR by using the PRO primer, as it has been shown that this captures most of the nucleotide variation along this region in red deer (Douzery and Randi 1997; Randi et al. 2001; Nussey et al. 2006). By this procedure, we obtained a sequence of 757 bp. Sequencing reactions were carried out in triplicate.

Fragment was sequenced using BigDye^™^ Terminator Cycle Sequencing Ready Reaction Kit, version 3.1 (Applied Biosystems, Foster city, CA). Sequencing reactions were purified using the Montage SEQ96 Sequencing Reaction Clean‐up Kit (Millipore, Billerica, MA) and analyzed in a DNA sequencer ABI PRISM 3130 (Applied Biosystems).

For nuclear loci, samples were genotyped with 15 microsatellite markers optimized in three multiplex PCRs and two capillary electrophoresis runs [MP1: CelJP15, MM12, OarFCB304, OarFCB193, ETH225, BM1818; MP2: OarFCB5, TGLA53, CelJP38; MP3: CSSM19, RME25, CSSM22, CSSM43, OarCP26, CSPS115 (Buchanan and Crawford 1993; Steffen et al. 1993; Bishop et al. 1994; Moore et al. 1994; Grosse et al. 1995; Ede et al. 1995; Mommens et al. 1994; Coulson et al. 1998]. DNA sequencer ABI PRISM 3130 and GENEMAPPER software (Applied Biosystems) were used to determine allele sizes.

### Analysis of mtDNA data

For mtDNA analyses, we used SeqScape v2.5 (Applied Biosystems) to visually inspect and correct sequences. Alignment was conducted using Bioedit (Hall 1999), and sequences were collapsed into haplotype with DNAsp software v.5.10 (Rozas et al. 2003). This software was also used to estimate haplotype (Hd) and nucleotide diversities (*π*).

To address the phylogeographic and evolutionary relationships among haplotypes, we carried out four types of phylogenetic analyses: (1) maximum likelihood (ML), (2) maximum parsimony (MP), (3) neighbor‐joining (NJ), and (4) Bayesian inference (BI). We used PAUP* 4.0b10 (Swofford 2002) with “tree‐bisection‐reconnection” (TBR) branch swapping under the optimality criteria ML and MP with random stepwise addition and NJ with model‐corrected maximum likelihood distances. Bootstrap analyses (Felsenstein 1985) were ran to test robustness of observed branching patterns with 1000 random repetitions for MP and NJ and 100 for ML. BI was implemented in MRBAYES 3.1 (Huelsenbeck and Ronquist 2001). Metropolis‐coupled Markov chain Monte Carlo sampling was performed with 10,000,000 iterations, sampled every 100 generations and using a burn‐in of 250 generations (determined by empirical checking of likelihood values via visual analysis of trace plots).

To determine the appropriate model of sequence evolution, we used the program MODELTEST 3.7 (Posada and Crandall 1998), which selects the best‐fit nucleotide substitution model using the likelihood ratio test—LRT—and the Akaike information criterion—AIC. The chosen model, K81uf+I+Γ (unequal frequency Kimura 3‐parameter, with gamma distributed rates and invariant sites), was applied to the data matrix to infer the phylogeography. Sequences of north African red deer (*Cervus elaphus barbarus*) (AF296807, AF296808), Sardinian red deer (*Cervus elaphus corsicanus*) (AF291885), Italian red deer (*Cervus elaphus hippelaphus*) (AF291886 and AF291887), Spanish red deer (*Cervus elaphus hispanicus*) (AF291889), and *Cervus elaphus* (U12867) were obtained from the NCBI nucleotide data bank and were included in phylogenetic analysis.

Gene flow (Fst) between groups was performed with DNAsp v5.10 (Rozas et al. 2003), gaps were excluded only in pairwise comparison, and permutation test was performed with 1000 replicates. Resulting distance matrix was used as input in Mega v3.1 (Kumar et al. 2004) in order to represent the results.

To estimate haplotype coalescence times, we applied a strict molecular clock with mutation rate 10.3% Myr‐1 according to Skog et al. (2009), where calibration of the mutation rate of D‐loop was made by comparing divergence in the D‐loop region with divergence in a coding region (cyt b) in pairwise comparisons of closely related deer species. We discarded a heterogeneous mutation rate among lineages checking the ucld.stdev parameter under a correlated relaxed lognormal clock as recommend by Drummond et al. (2012). Divergence time for the different haplogroups was estimated by using the Bayesian coalescence approach implemented in BEAST v.1.4.5 (Drummond et al. (2012). Each split between haplogroups was estimated using Markov chain Monte Carlo (MCMC) simulations for 10,000,000 generations, with the first 10% discarded as burn‐in. Convergence and results were checked using the program TRACERv1.5 (Rambaut et al. 2014).

### Microsatellite data analysis

Genetic diversity was quantified as observed heterozygosity (*H*_*o*_), expected heterozygosity (*H*_*e*_), and mean number of alleles per locus (*A*) with Genetix version 4.05 (Belkhir et al. 2004).

We applied the Bayesian methodology implemented by STRUCTURE version 2.0 (Pritchard et al. 2000) to determine the level of genetic substructure in our data independently of the known origin of individuals. To determine the number of genetic clusters (*K*), 10 independent runs of *K *=* *1–9 were carried out with 50,000 iterations, following a burn‐in period of 1,000,000 iterations. We assumed admixture and a correlation among genetic frequencies. We used an ad hoc statistic *ΔK* to identify the true number of genetic clusters (*K*) (Evanno et al. 2005). The 10 runs of the true *K* value were averaged using CLUMPP version 1.1.1 (Jakobsson and Rosenberg 2007) and displayed by DISTRUCT version 1.1 (Rosenberg 2004). After a first run that identified the major genetic discontinuities, we made second runs separately with each genetic group obtained in the first run. Thus, we looked for minor genetic discontinuities within major genetic areas.

In order to check for structure in our data with different approaches, firstly pairwise *F*_*st*_ values between the genetic clusters obtained with STRUCTURE were quantified using SPAGeDI version 1.3 (Hardy and Vekemans 2002). PHYLIP 3.5 (Felsenstein 2005) was used to construct an unrooted neighbor‐joining tree from pairwise *F*_*st*_ values. Additionally, we ran a factorial correspondence analysis (FCA) for all populations with GENETIX.

Finally, we compared the geographical distance and genetic relatedness between all possible pairs of individuals in our populations with SPAGeDI. We considered as the geographical location of individuals the mean point of the area each population represents. For the analyses, we used the genetic relatedness coefficient described in Wang (2002). To assess whether the degree of genetic relatedness between individuals depended on the geographic distance, we carried out a permutation test. In this test, spatial locations were permuted among the available locations to test the regression slope. We used 10,000 permutations.

## Results

### Sequence variation and genetic diversity

The mtDNA CR sequence alignment (757 bp including sites with gaps) of 682 deer samples was collapsed in 45 haplotypes (GenBank accession nos KT202236‐KT202280), defined by 61 polymorphic sites (8.1%) of which 44 (5.8%) were parsimony informative. Base composition was biased to a deficiency of guanine (A = 29.0%; C = 23.6%; T = 30.3%; G = 16.9%).

Genetic diversity indices for the red deer populations analyzed in this study are summarized in Table 2. Despite *C. e. hispanicus, scoticus,* and *atlanticus* presented similar values of haplotype diversity (Hd ≅ 0.8), *C. e. hispanicus* showed the highest nucleotide diversity value (*π* × 100 = 0.999), indicating a great amount of divergence between haplotypes within Iberian (Spanish) red deer. The lowest values of genetic diversity were found in the sampled population of *C. e. elaphus* (Hd = 0.255 and *π* × 100 = 0.108).

**Table 2:** Genetic diversity indices for the red deer populations (from the 45 haplotypes found in the 682 samples). *N*: number of sequences; *S*: number of polymorphic sites; *h*: number of haplotypes; Hd: haplotype diversity; Π × 100: nucleotide diversity × 100. Spanish samples are also shown separately for the two main lineages, see below (central, Sc, and west, Sw)

Population	*N*	S	h	Hd (SD)	Π × 100 (SD)
*Cervus elaphus hispanicus (Sc)*	501	27	19	0.797 (0.012)	0.852 (0.013)
*C. e. hispanicus (Sw)*	110	10	6	0.734 (0.017)	0.536 (0.023)
*C. e. hispanicus* Total	611	36	25	0.855 (0.009)	0.999(0.009)
*C. e. elaphus*	22	8	3	0.255 (0.116)	0.108 (0.001)
*C. e. scoticus*	20	16	7	0.816 (0.058)	0.524 (0.001)
*C. e. atlanticus*	21	4	5	0.824 (0.037)	0.204 (0.000)
*C. e. hippelaphus*	8	8	3	0.464 (0.200)	0.265 (0.002)

Most haplotypes were unique among subspecies. Among the 45 haplotypes found, only two were sampled in more than one subspecies: H19 was sampled in both *C. e. scoticus* and *C. e. atlanticus* distribution ranges and H6 that was very common in southern Spain and also corresponded to the haplotype identified in a trophy from Germany dated to 1872. In our sample, haplotype H6 was the most frequent (28%), although it was mostly restricted to Sierra Morena (Hornachuelos, Córdoba province), followed by H16 and H9 (12 and 9%, respectively).

### Phylogeographical relationships

**Figure 1:**
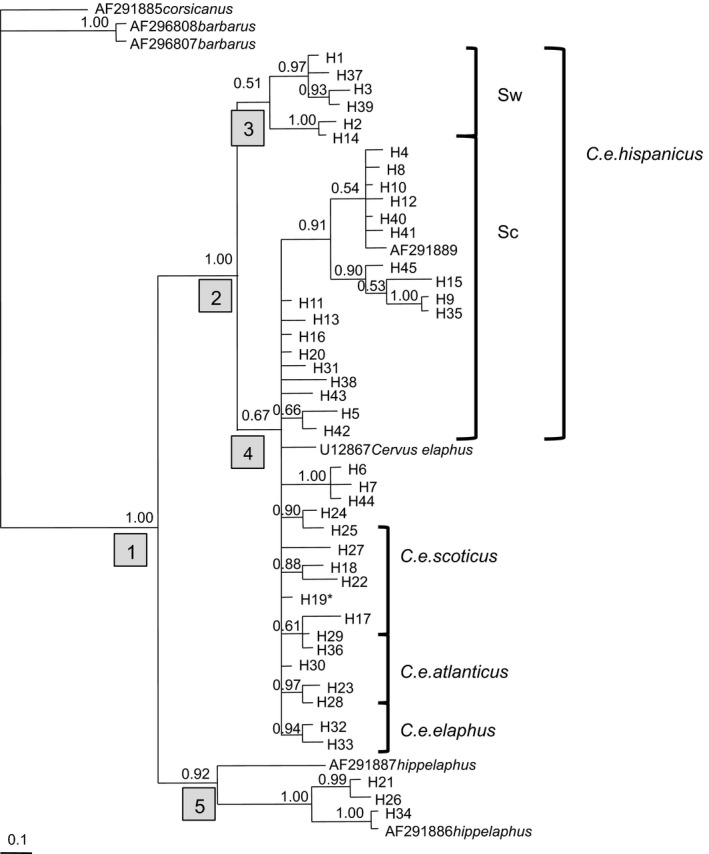
Phylogenetic reconstruction of the 45 red deer haplotypes found in this study (*n* = 682) from the 757 bp of the control region (D‐loop) analyzed. Branch lengths are Bayesian posterior probabilities and the scale bar represents the mean number of nucleotide changes per site. Main nodes labeled with numbers are used below for estimating divergence times (Table 4).

The statistically appropriate model for the data set with outgroups was K81uf+I+G. This evolutionary model was selected by Akaike information criterion (AIC), which has been more precise than the hierarchical likelihood ratio test (Posada and Buckley 2004). The four types of phylogenetic analyses produced consensus trees with a similar topology (not shown) with three major clusters easily identified. The earlier differentiated cluster rooting the tree (BI tree) corresponded to clade B in Ludt et al. (2004) with lineages of north Africa and Sardinia (see Fig. 1). A second clade contained *C. e. hippelaphus* sequences from GenBank and individuals sampled in Italy, where it is known that populations were restocked with *C. e. hippelaphus* (Randi et al. 2001), thus corresponding with the Eastern red deer, clade C in Ludt et al. (2004). The other cluster corresponds to the Western red deer, clade A in Ludt et al. (2004).

**Figure 2:**
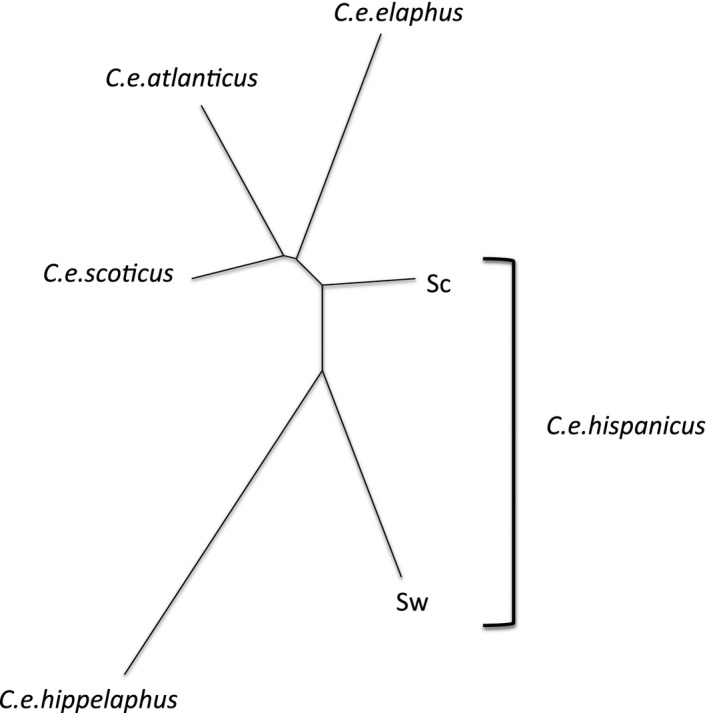
Genetic pairwise differentiation between red deer assemblages based on mtDNA haplotypes. See Table 3 for Fst values and significance.

Within the western lineage, two clusters became consistent in all consensus trees, although with low bootstrap values (0.51 and 0.67, respectively, for both clusters in Fig. 1). The first cluster corresponded to samples from western Spanish populations (Sw), while the other contained the remaining sampling sites, including central‐south Spanish (Sc) and north European populations (Norway, Scotland, and Germany). Within this second cluster, the large number of haplotypes and the relatively small number of variable sites limited the resolution of the phylogenetic analysis showing many polyphyletic relationships. Most groupings were only formed by two or three haplotypes, with the exception of one subclade sampled in central Iberian Peninsula (with bootstrap value 0.91 within Sc haplogroup) with most internal relationships highly resolved (Fig. 1). Pairwise genetic differentiation between haplogroups supported the relationship between Iberian Sc cluster and northern European populations (Table 3, Fig. 2).

**Table 3:** Pairwise Fst values based on haplotype frequencies. Significance was evaluated by permuting haplotypes among samples (1000 permutations). All pairwise comparisons were statistically significant (*P* < 0.001)

	*Cervus elaphus hispanicus (Sc)*	*C.e.elaphus*	*C.e.scoticus*	*C.e.atlanticus*	*C.e.hippelaphus*
*C.e.elaphus*	0.492				
*C.e.scoticus*	0.284	0.450			
*C.e.atlanticus*	0.421	0.512	0.333		
*C.e.hippelaphus*	0.667	0.860	0.695	0.829	
*C.e. hispanicus (Sw)*	0.448	0.690	0.522	0.602	0.725

**Figure 3:**
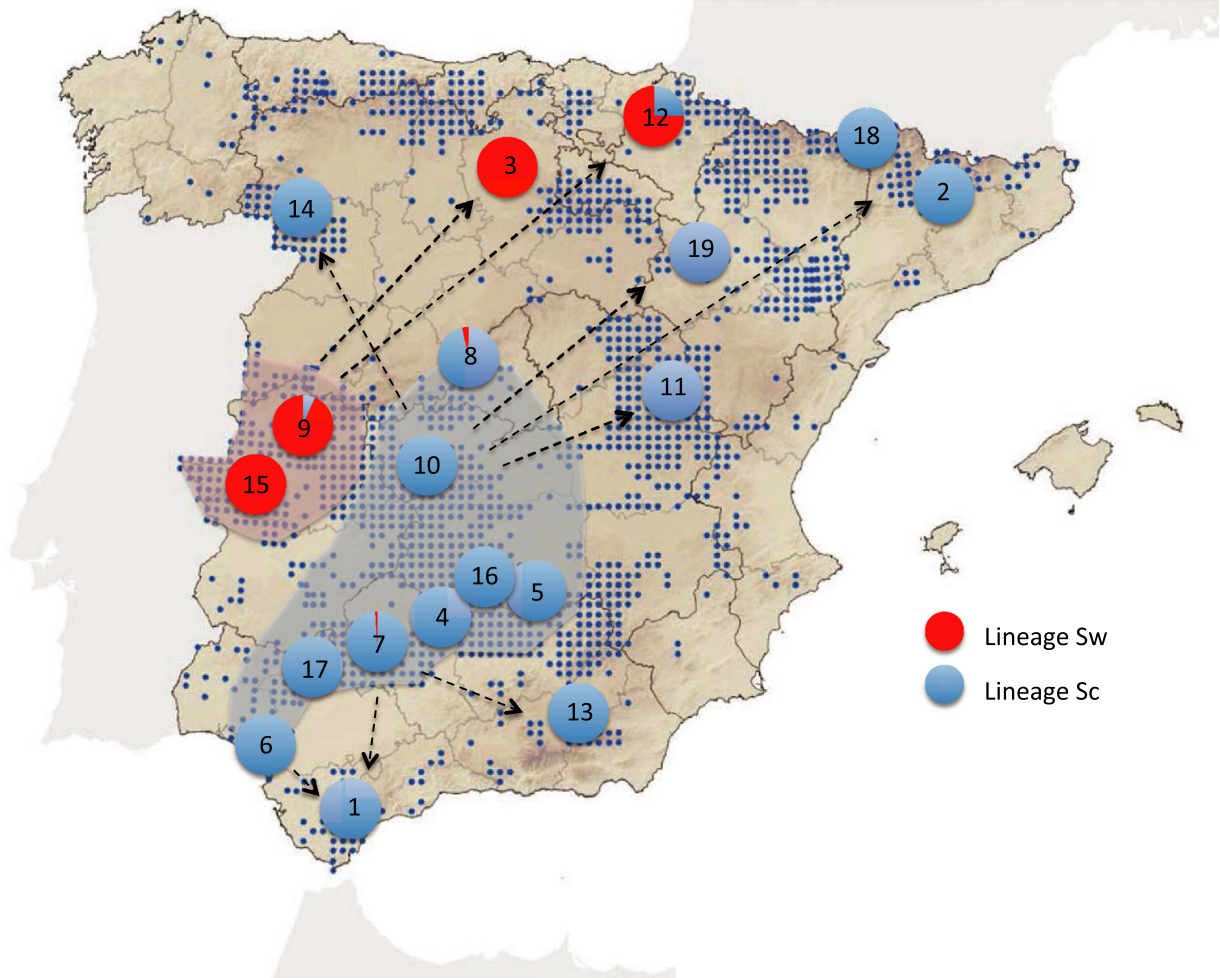
Sampling sites and Iberian clusters: Map shows current distribution of red deer in Spain (dots indicate presence in UTM 10 × 10 km, after Palomo et al. 2007) and location of sampling localities indicated with numbers that correspond to those appearing in Table 1. Circles at each location depict the relative frequency of samples from both haplogroups. Shaded areas show possible natural distribution ranges for both haplogroups, while arrows indicate known restocking routes (information on restocking after Pérez et al. 1998; Carranza 2011, and J. Carranza unpublished data).

Figure 3 shows the distribution of Sc and Sw haplogroups in Spain, along with a classification of populations into putative natural distribution ranges and restocked sites (based on Pérez et al. 1998; Carranza 2011; J. Carranza unpublished data). Current red deer populations in most areas of north, northeast, and southeast were founded by restocking since the mid 20th century, with specimens from the two main areas that maintained native remaining populations (shaded areas in Fig. 3). Sampled populations in western Spain belonged almost exclusively to Sw haplogroup, and there is evidence (J. Carranza unpublished) that the two populations sampled in north Spain with Sw haplotypes were restocked from the western area.

### Divergence times

On the basis of a mutation rate of 10.3% Myr^−1^ (Skog et al. 2009), our results indicate that eastern European (node 5) (haplogroup A in Skog et al. 2009) and western European (node 2) lineages diverged at an estimated time of 227 Kyr before present. The mean and 95% highest posterior density interval for each divergence time are indicated in Table 4.

**Table 4:** Bayesian estimates and 95% credibility intervals of red deer phylogroups divergence times estimated using BEAST. See Fig. 1 for positions of nodes in the phylogenetic tree

Calibrated node	Mean age of prior (Kyr)	Standard deviation	Credible interval on age prior (Kyr)	
			Lower	Upper
1	227	1.738	154	314
2	109	1.073	69	150
3	88	0.526	54	127
4	76	0.919	24	131
5	156	1.626	83	231

Within West European red deer, the earliest divergence occurred between west Iberian lineage and the remaining haplogroups, with an estimated time of 109 Kyr (node 3) before present for the coalescence of the ancestors of both branches. Within the haplogroup that includes central‐south Iberian and north European populations, the main central‐south Iberian clade was inferred to diverge at approximately 88 Kyr (node 4), while the remaining central‐south Iberian and north European lineages diverged at approximately 76 Kyr. Thus, the phylogenetic pattern for Western European red deer indicates a deep division dated at 109 Kyr that differentiated both Iberian lineages, and subsequent differentiation events within these clusters that were dated at 88 and 76 Kyr, giving rise to the radiation within central‐south Iberian–north European main haplogroups and within the west Iberian lineage, respectively.

### Neutrality test and demographic history

**Figure 4:**
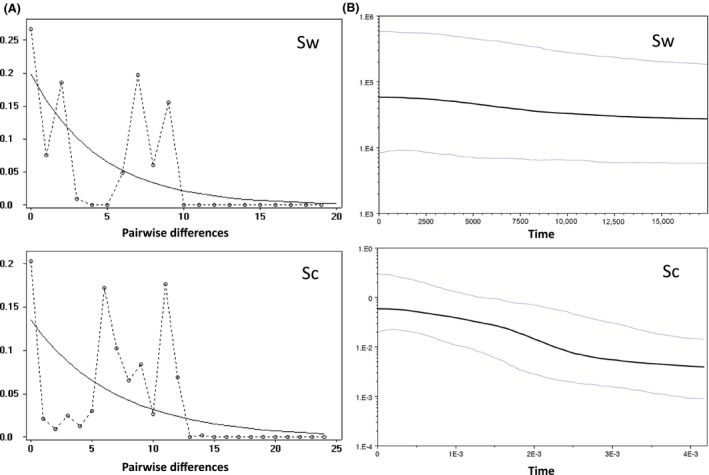
(A) Mismatch distribution of mtDNA haplotypes for each of the Spanish red deer phylogroups. The expected frequency is based on a population constant model and is represented by a continuous line. The observed frequency is represented by a dotted line. (B) Bayesian skyline plots showing the historical demographic trends for each of the Spanish red deer phylogroup. The *y*‐axis express population size, estimated in units of Ne*μ* (Ne: effective population size, *μ*: mutation rate per haplotype per generation) in a log‐scale. Median estimates and confidence intervals are shown.

Significant deviation from neutrality was detected only within the phylogroup Sw with Tajima's D (2,84**) but significance disappeared when more powerful statistic was applied (Fu's Fs: 6.84 and 4.45 for Sc and Sw, respectively, and 0.2 and 0.11 for R2) using DNAsp software (Rozas et al. 2003). The distributions of pairwise differences within each phylogroup were also consistent with population samples at demographic equilibrium, with steep waves and a high frequency of diverged haplotypes (Rogers and Harpending 1992). Historical demographic reconstructions (BSPs) also showed equilibrium trends, but both lineages differed in their demographic history, with Sc showing greater population expansion (Fig. 4).

### Genetic structure at microsatellite markers

**Figure 5:**
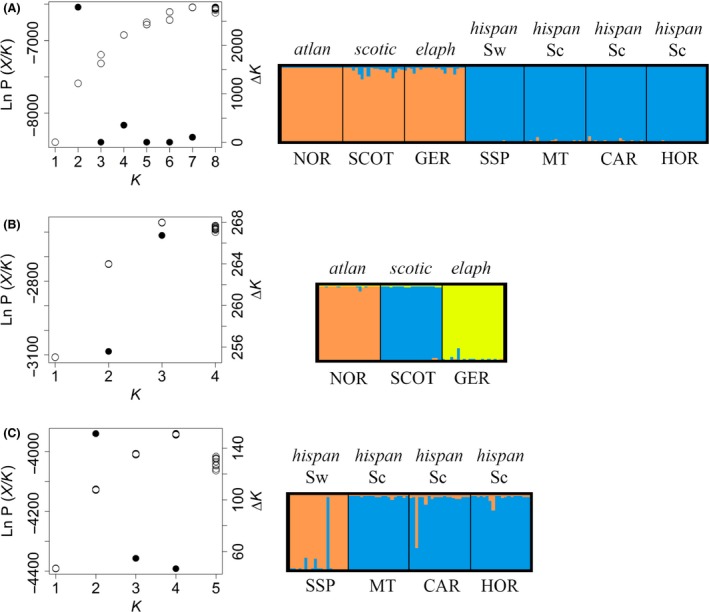
STRUCTURE software results. (A) All populations. Graph shows the log‐likelihood values, Ln Pr (*X*⁄*K*) (white points) and Ad hoc statistic *ΔK* (black points) showing the most probable number of genetic clusters (*K*). Membership coefficients of probability for each cluster are also showing. (B) Non‐Spanish populations. (C) Spanish populations. NOR: Norway; SCOT: Scotland; GER: Germany; SSP: Sierra de San Pedro; MT: Montes de Toledo; CAR: Cardeña; HOR: Hornachuelos. Different subspecies are coded with the following notation: *atlan*:* C. e. atlanticus; scotic: C. e. scoticus; elaph: C. e. elaphus; hispan: C. e. hispanicus*.

**Figure 6:**
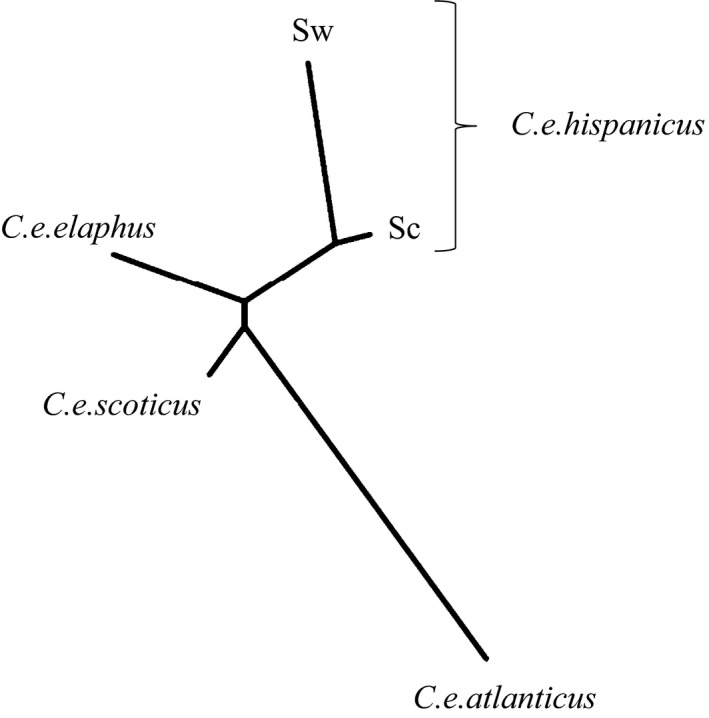
Unrooted neighbor‐joining tree for the obtained genetic clusters (Norway, Scotland, Germany, west Spain, central‐south Spain) based on microsatellite allele frequencies.

**Figure 7:**
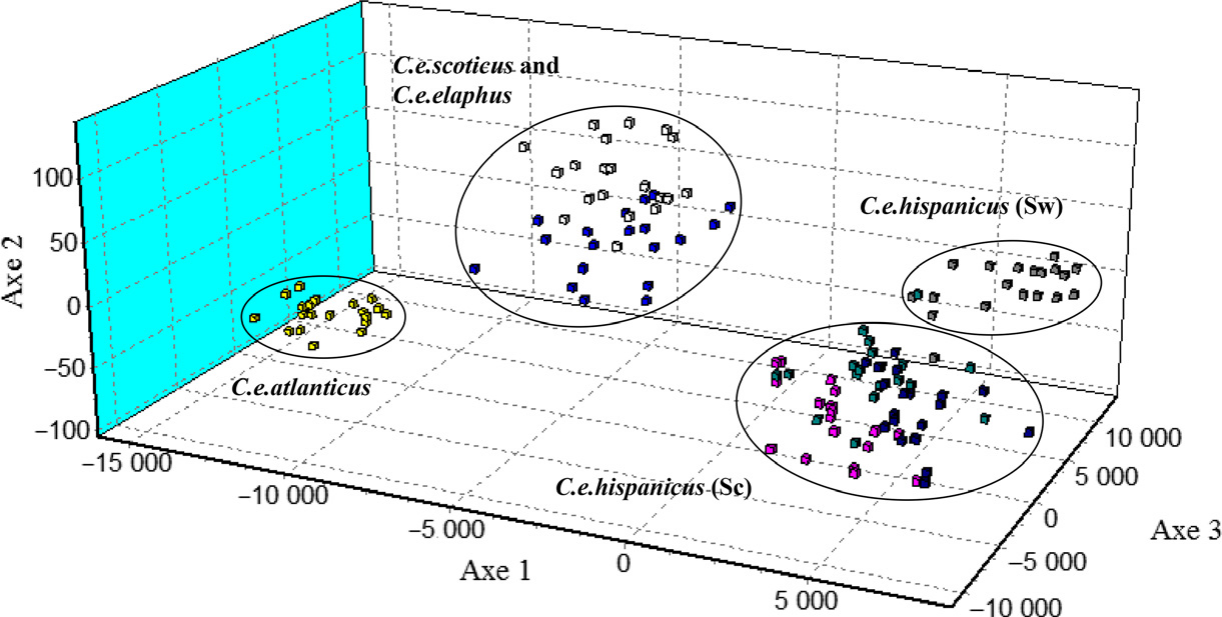
Three‐dimensional plot of the factorial correspondence analysis (FCA). Populations are coded with different colors: Sierra de San Pedro (gray), Montes de Toledo (pink), Cardeña (green), Hornachuelos (dark blue), Norway (yellow), Scotland (blue), and Germany (white). To facilitate interpretation, we indicate different areas in the plot with circles. Individuals from Scotland and Germany are mixed in the same space. One individual from Sierra de San Pedro appears within the central‐south Spain area and one from Cardeña in west Spain.

Bayesian methodology implemented by STRUCTURE showed that *K *=* *2 was the most probable number of clusters (Fig. 5A). Plotting individual probabilities for each *K* value revealed two population sets, one of them including non‐Spanish populations and the other including Spanish populations (Fig. 5A). In the second run using non‐Spanish populations, *ΔK* was maximized in *K *=* *3 (Fig. 5B). Individual probabilities for each *K* value coincided with the three populations: Norway, Scotland, and Germany (Fig. 5B). In the second run using Spanish populations, *K *=* *2 was the most probable number of clusters (Fig. 5C) and two population sets were revealed: west Spain (San Pedro) and central‐south Spain (Montes de Toledo, Cardeña, and Hornachuelos; Fig. 5C). In this second run, some individuals appeared assigned to populations different from those in which they were sampled (Fig. 5C): one male in Sierra de San Pedro (west Spain) with more than 0.9 membership coefficient for the other group (central‐south Spain), and one male in Cardeña (central‐south Spain) with more than 0.7 membership coefficient for west Spain. Table 5 shows the genetic diversity measures for each sampled population and for the different clusters of populations after STRUCTURE runs. Figure 6 shows the genetic distances (pairwise *F*_*st*_ values) between the genetic clusters identified by STRUCTURE analyses. Figure 7 shows a three‐dimensional plot of the factorial correspondence analysis (FCA).

**Table 5:** Genetic diversity at microsatellite markers. *H*_*e*_: expected heterozygosity, *H*_*o*_: observed heterozygosity and *A*: mean number of alleles per locus. (a) Genetic diversity for each population. (b) Populations grouped in the clusters obtained with STRUCTURE in the first run. (c) Populations grouped in the clusters obtained with STRUCTURE in the second run. See text and Figure 5 for details

	*H*_*e*_	*H*_*o*_	*A*
(a) Sampled populations			
Sierra de San Pedro (west Spain)	0.674	0.636	6.000
Montes de Toledo (central‐south Spain)	0.732	0.713	8.071
Cardeña (central‐south Spain)	0.719	0.647	7.333
Hornachuelos (central‐south Spain)	0.774	0.736	8.200
Norway	0.549	0.446	4.200
Scotland	0.703	0.590	7.400
Germany	0.681	0.650	6.333
(b) Clusters from STRUCTURE first run			
Spain	0.805	0.681	12.067
North Europe	0.753	0.563	10.466
(c) Clusters from STRUCTURE second runs			
West Spain	0.674	0.636	6.000
Central‐south Spain	0.801	0.694	11.400
Norway	0.549	0.446	4.200
Scotland	0.703	0.590	7.400
Germany	0.681	0.650	6.333

**Figure 8:**
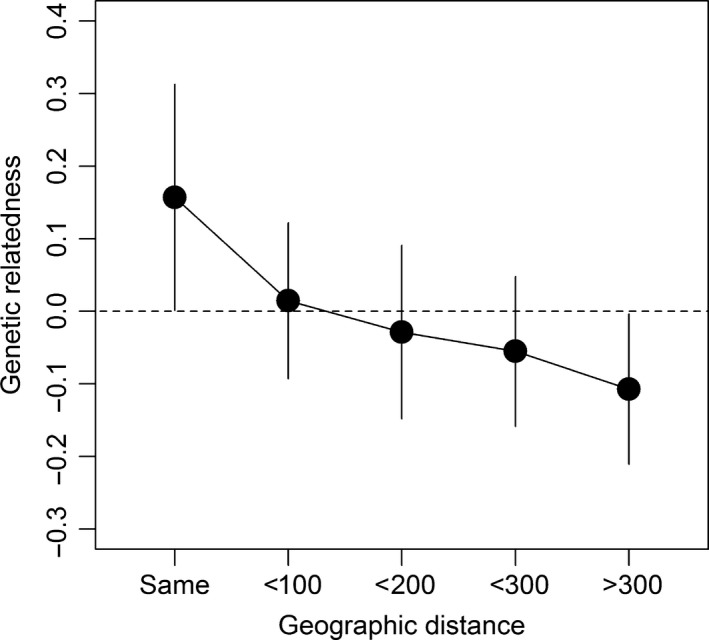
Relationship between geographical distance and genetic relatedness between individuals. Average estimates were taken for individuals separated in the following distance intervals: Same population; <100 km (individuals from Cardeña and Hornachuelos were compared); <200 km (individuals from Montes de Toledo‐Cardeña and Montes de Toledo‐Hornachuelos were compared); <300 km (individuals from Sierra de San Pedro‐Montes de Toledo, Sierra de San Pedro‐Cardeña and Sierra de San Pedro‐Hornachuelos were compared); >300 km (individuals from different countries were compared). Figure shows mean and standard deviation.

Genetic relatedness between pairs of individuals declined with geographic distance (10,000 permutations, slope with the linear distance = −0.00006, slope with the logarithmic distance = −0.025, *P* < 0.001; Fig. 8).

## Discussion

Our results indicate that the genetic relationships within West European red deer are informed differently depending on whether we take into account mitochondrial or nuclear DNA: The differentiation of Iberian red deer (classical Iberian subspecies) can be supported on the basis of nuclear DNA, while mtDNA uncovers for the west European clade a differentiated refugial lineage for western Spain populations.

Phylogenetic differentiation of red deer in Europe based on mtDNA assumes three primary clades (A, B, and C in Skog et al. 2009) with strong geographic clustering, one of them (C) in the Balkans and Caucasia, another (B) Corso‐African red deer, and (A) in west Europe (see also Sommer et al. 2008), for which each glacial refugium left historic genetic signatures in every native stocks (Hewitt 2004). However, it has also been acknowledged that a largest genetic subdivision should be expected because the (three) surviving European stocks come from an undetermined number of isolated refugium pockets at southern Europe (Sommer and Nadachowski 2006; Schmitt 2007; Hajji et al. 2008). Our results after a stronger sampling effort in Spain compared to previous studies indicate that the western European lineage probably splits into two matrilineal branches during the Würm glacial period within the Iberian refugium (in agreement with Fernández‐García et al. 2014) and that only one of them (Sc) contributed to the extant populations in northern areas of western Europe. The highest nucleotide diversity of this central‐south Iberian haplogroup (Sc) supports its basal (ancestral) position in the phylogeographic divergence of western European lineages. This finding refines the results of Skog et al. (2009) for the west European clade (A), pointing to the central‐south Iberian haplogroup (Sc) as the source of most haplotype diversity in this lineage. Thus, our data do not support an eventual ancestral nature of Norwegian populations as suggested by Skog et al. (2009). Also, we did not find haplotypes belonging to the lineage occurring in northern Africa and Mediterranean isles in natural areas of Spain, contrarily to the finding of Skog et al. (2009). One possible interpretation is that the single sample of Sardinian haplotype sampled in Spain by Skog et al. (2009) may come from recent restocking. Lineage B (north Africa and Sardinia) matrilines have been the subject of many introductions and farm rearing, especially in Britain (Carden et al. 2012), so haplotypes of this group are likely candidates to appear in translocated stocks coming from farms. This would explain the sampling of this type of Corso‐African haplotypes in Rum Island (Nussey et al. 2006) or from a captive red deer in Germany (Ludt et al. 2004).

Therefore, our results indicate that Iberian red deer (*Cervus elaphus hispanicus* Hilzheimer 1909) may be a paraphyletic subspecies that encompasses two genetic lineages that predated the last glacial maximum with one of them leading to different subspecies during the Holocene when recolonized northern areas in Europe. The genetic differentiation of the western Iberian lineage (Sw) was not strongly supported on the basis of the mtDNA phylogenetic tree, but the topology splitting both haplogroups within Spain was consistent in all consensus trees from all four methods used, while the central‐south Spanish populations showed very low genetic differentiation with respect to north European populations at mtDNA sequences. For nuclear DNA, however, the West European red deer was firstly split into two different groupings: Iberian and non‐Iberian populations. In the search for lower genetic discontinuities, the Iberian populations appeared then divided into two clusters corresponding to western and central‐south Iberian haplogroups.

Historical and demographic processes acting over West European red deer during the Pleistocene may have produced different signatures in each type of DNA markers. Paleontological information (Sommer et al. 2008; Meiri et al. 2013) indicates that Iberian and northern European populations may have separated as recently as 11,000 years ago. Mitochondrial DNA regions (cyt b, D‐loop) may change between 2–10% per million years (see e.g., Hewitt 1996; Pitra et al. 2004; Peters et al. 2005; Weir and Schluter 2008), so we may expect at most 0.03–0.16% nucleotide substitutions during postglacial recolonization (i.e., ca. a single base substitution for the whole D‐loop sequence). By contrast, microsatellites have much higher mutation rates (e.g., Sun et al. 2012) producing large numbers of alleles that can also produce large variations in frequencies among populations within a short period of time (Hewitt 1999).

Our results evidenced at least three remarkable facts. On the one hand (first subheading below), mtDNA indicates an earlier differentiation during Würm glacial times in the Iberian Peninsula that maintains signatures in current populations at two main geographical areas in Spain, while postglacial recolonization of northern Europe has not produced noticeable differentiations at the level of mtDNA with respect to the Iberian refugial lineage to which they belong. Meanwhile, nuclear loci have revealed two additional facts. One of them (second subheading below) is that genomes of north‐expanding populations differentiated not only from their Iberian ancestors (haplogroup Sc) but also among themselves, especially affecting the northernmost Norwegian populations. Red deer might have disappeared at these northern ranges during the LGM or might have maintained some small stocks, but it seems most probable that all current native populations in northwestern Europe descend from the postglacial recolonization from the Iberian refugium (Meiri et al. 2013). But also (third subheading below), it is also remarkable that the two regions in Spain that maintain a notably high differentiation at the level of mtDNA haplotypes appear quite closely related at the level of microsatellite loci, and more related between them than with northern European populations, in agreement with the subspecific subdivisions based on phenotypic features.

### Mitochondrial DNA differentiation within Iberia

Although bootstrap support for Sw haplogroup was low (0.51), it evidenced a genetic structure within Iberian red deer deeper than with distant populations in northern Europe. Haplotype diversity was lower for group Sw, but the phylogenetic relationships do not support a recent origin of Sw from the other Iberian lineage Sc. Rather, molecular divergence times indicate a differentiation that predated the LGM, which points to two refugial areas within Iberia. There are many examples of genetic differentiation at the level of mtDNA within Iberia during the Pleistocene for diverse taxa, from invertebrates to all vertebrate groups and many species of plants (see the reviews by Gómez and Lunt 2007; Arroyo et al. 2004; Schmitt 2007) evidencing the existence of several refugia within the Iberian refugium (Gómez and Lunt 2007). An interesting similar pattern to the one found here for red deer was reported for the field vole (Jaarola and Tegelström 1995; Jaarola and Searle 2002). The southern mtDNA lineage of this rodent was postulated to have recolonized southern France, Switzerland, Slovenia, and Hungary from an Iberian refugium (Jaarola and Searle 2002). Detailed sampling in the Iberian Peninsula revealed two lineages, one of them restricted to Serra da Estrela in Portugal (M. Jaarola & J. B. Searle, pers. comm.). The authors hypothesized that the field vole survived at least through the last glaciation in two Iberian refugia. A recent study with the red‐legged partridge (*Alectoris rufa*) found five genetic clusters evidencing three glacial refugia within the Iberian refugium (Ferrero et al. 2011).

Paleoclimatology shows that the climatically most favorable areas of Iberia during the last glaciation were in disjunct areas in the southwestern and the southeastern parts of the peninsula (Herterich 1991). Main Iberian refugia during the LGM might have been located near the extreme SW and SE coastal areas at the Atlantic and the Mediterranean coastlines, respectively (Herterich 1991; Branco et al. 2002; Gómez and Lunt 2007; Miraldo et al. 2011). The putative location of these two refugia suggests a possible scenario of divergence and postglacial colonization for Iberian red deer lineages. Populations at the Sw refugium would have expanded northwards to Extremadura and possibly Portugal, although we lack native samples from Portugal. Populations at the SE refugium would have expanded to Central Spain and northwards through France to northern European areas including Britain and Scandinavia. These results open new questions on why populations from SE refugium expanded to the extreme north of Europe but hardly merged with the populations in western Iberia (at least for the matrilines). One possible explanation is that already occupied areas may prevent the expansion of matrilines compared to newly available ranges in the north (Hardin 1960). For dispersing males, however, it is more likely that some individuals could have moved across the hybrid zone (Clutton‐Brock et al. 1982, 2002; Catchpole et al. 2004; Nussey et al. 2005; Frantz et al. 2008), which might explain the similarities found at nuclear loci (see below).

The ranges of current native populations (i.e., excluding restocked populations) of Iberian lineages in western (Sw) and central‐southern Spain (Sc) show a secondary contact zone located in NE‐SW direction at the mountain range occurring in eastern Extremadura (Villuercas and Sierra de Pela). Contact zones are common for other taxa in Iberia, although the location and direction are highly variable (e.g., rabbits *Oryctolagus cuniculus*: Branco et al. 2002; *Lacerta lepida*: Miraldo et al. 2011; see rev. Gómez and Lunt 2007).

### Genetic differentiation at nuclear loci during postglacial recolonization

Northwestern European populations are mostly descendants of expansions and recolonization of dispersal individuals from Iberia (Meiri et al. 2013), in particular from the south‐central Iberian lineage (our mtDNA results). Our data for allele diversity agree with this pattern. Genomes may change very rapidly when subjected to range expansion (Excoffier et al. 2009; Short and Petren 2011). Range changes during postglacial recolonization may be characterized by leptokurtic expansion leading to loss of alleles and large patches of homozygosity (Hewitt 1999). Demographic events such as founder effects or bottlenecks might generate genetic differentiation due to the action of genetic drift (Hewitt 2004). These demographic and genetic processes seem to have been extreme for the Norwegian population that presented the highest values of genetic differentiation within the non‐Spanish clade (see also Haanes et al. 2011).

The period of time is not short for changes in allele frequencies. An increasing number of papers show how changes in the species distribution ranges, either related to invasive species or as effects of climate change, are leading to significant changes in allele frequencies within short periods of time (e.g., Short and Petren 2011; Chown et al. 2014). Furthermore, demographic and domestication processes on large mammals, such as for instance the case of dogs (Thalmann et al. 2013; Freedman et al. 2014), have also demonstrated that nuclear loci changed very rapidly (within a period of less than 15,000 years, hence very similar to our case for red deer during postglacial recolonization) and produce such strong genomic (and phenotypic) departures from their ancestors (Freedman et al. 2014), despite mtDNA maintained a completely different pattern by which dogs appeared more related to the several wolf matrilines from which they descend than among themselves (Thalmann et al. 2013).

### Gene flow between Iberian stocks

Our analyses with nuclear loci show that Spanish populations, regardless of mtDNA lineages, constituted a genetic group different from other western European populations. This result agrees with the phenotypic features typical of Iberian red deer populations of either lineage that contrast with northern subspecies, affecting morphology (Geist 1998), mating behavior (Carranza et al. 1990), anatomy (Frey et al. 2012), and acoustic features of male roars (Passilongo et al. 2013). These similarities might have arisen by phenotypic plasticity (Geist 1998) or by evolutionary convergence (see Duarte et al. 2008 for a deer example) when adapting to the environment of the Iberian Peninsula, and we cannot rule out a potential role of these types of processes. However, our results at nuclear genetic markers show that homogeneity of Iberian red deer might not be the consequence of plasticity or evolutionary convergence. There is evidence that genetic flow due to male dispersion may homogenize genomic dissimilarities between previously differentiated maternal lineages (see e.g., Yang and Kenagy 2009; Rodríguez et al. 2010). The persistence of different mtDNA lineages at both areas in the Iberian Peninsula supports a low interchange of individuals between both Iberian refugia since the last glacial maximum. Because of mtDNA is only inherited from females, interchange of haplotypes between populations can only be effective with female dispersal (see Prugnolle and de Meeus 2002). In natural populations, red deer has a male‐biased dispersal in which females normally remain in the areas where they were born (Clutton‐Brock et al. 1982; Pérez‐González and Carranza 2009). Gene flow by male dispersal might have occurred from the last glacial maximum to present, and we cannot reject the maintenance of some male dispersal between areas during climate oscillations of the long‐lasting Würm glaciation. Additionally, genetic differentiation might be reduced by the existence of large effective population sizes in Iberian refugia (Falconer and Mackay 1996) compared to the differentiation of populations that migrated to north Europe during postglacial recolonization.

Nowadays, barriers that could divide Iberian populations into two isolated groups are lacking. However, during the last glacial maximum, environmental conditions must have been unfavorable in the central plateau of Iberia as to maintain isolation between coastal refugial areas (Herterich 1991) thus leading to the relatively high frequency of taxa with two or more different genetic lineages within Iberian Peninsula (e.g., Pérez‐Suárez et al. 1994; Comes and Abbott 1998; Branco et al. 2000; Lumaret et al. 2002). Male‐biased dispersal, along with the existence of barriers between populations (Pérez‐Espona et al. 2008; Pérez‐González et al. 2012), might have favored the genetic isolation of both Iberian lineages regarding mtDNA haplotypes along with the differentiation of Iberian red deer from northern populations.

Gene flow between Iberian lineages may have increased in recent times, either because of the increased size of populations and their geographic ranges during the last century in Spain (Carranza 2010), or by artificial translocations under the rise of hunting management in the last three or four decades (Martínez et al. 2002; Carranza et al. 2003; Carranza 2010), which may also be oriented to males. In fact, our analysis has detected some individuals (males) that did not assign to the genetic cluster to which their sampling population belonged, suggesting recent dispersal or translocations. However, evidence indicates that these recent movements alone cannot explain the homogenization of Iberian red deer. On the one hand, phenotypic features suggest a deeper homogenization of Iberian populations. But also, the isolation‐by‐distance pattern found here for Iberian red deer is more compatible with a natural gradient of gene flow between neighboring populations than with artificial translocations.

## Concluding remarks

Most papers on European red deer phylogeography have used only mtDNA (Ludt et al. 2004; Skog et al. 2009), while some studies on particular populations used both microsatellites and mtDNA (Zachos et al. 2003; Feulner et al. 2004; Hmwe et al. 2006). It is increasingly clear that we need both mitochondrial and nuclear markers to understand phylogeographic patterns in species with male‐biased dispersal and especially when looking at changes that took place mainly during the Holocene.

Our results with mitochondrial and nuclear DNA provide different, complementary information to the phylogeographic pattern of west European red deer. Sequences of the control region of mtDNA show that the red deer western European lineage splits into two matrilineal branches during the Würm glacial period within the Iberian refugium, and that only one of them (Sc) contributed to the extant populations in northern areas of western Europe. Along with morphology (Geist 1998), behavior (Carranza et al. 1990; Passilongo et al. 2013) and anatomy (Frey et al. 2012), microsatellite data support the idea of a homogeneous Iberian red deer group. Despite the existence of two mtDNA lineages in the Iberian Peninsula, as well as two probable refuges during the last glacial maximum, Iberian red deer can be considered a group genetically different from other western European populations. Iberian red deer is currently considered a subspecies (*C. e. hispanicus* Hilzheimer 1909) differentiated from the other red deer subspecies, and in particular from the west European subspecies (*scoticus, elaphus, atlanticus)*, on the basis of morphology (Geist 1998). Data from nuclear loci support this morphological singularity in the west European context although reveal that it originated from two distinct matrilines leading to a paraphyletic group (Crisp and Chandler 1996).

A debate exists in taxonomy on considering paraphyletic entities as real taxa (Nordal and Stedje 2005; Brummitt 2006; Hörandl 2006; Rieppel 2006). To some authors, only actual clades should be considered as taxa (Ebach et al. 2006). Other authors, however, advocate for using explicit phylogenetic information to accompany taxonomical decisions and maintain paraphyletic taxa when appropriate (Crisp and Chandler 1996; Brummitt 2006). Indeed, paraphyly is very common along phylogenies and most major taxa are in fact paraphyletic (see Funk and Omland 2003; McKay and Zink 2010; Ross 2014). Alternatively, paraphyletic entities might be split into their monophyletic constituents (Kristensen 1982). In our case, we might differentiate the two Iberian clades as subspecies along with the other north European subspecies. However, both Iberian lineages share important similarities that probably are not homoplasies but the result of either the conservation of common features or gene flow thanks to geographical proximity. Thus, we prefer to leave the assessment for the taxonomists, and here simply reveal the phylogenetic relationships and the likely evolutionary history of the group.

Another issue concerns conservation. To which extent we should rely on mitochondrial or genomic DNA to define evolutionary significant units (ESU) may be debatable (Moritz 1994; Crandall et al. 2000; Wan et al. 2004), but it is increasingly clear that we should use a suite of different genetic markers to understand the processes and decide on their relevance for preservation (Fraser and Bernatchez 2001). Restocking with foreign deer is still common in current red deer populations in spite of legal regulations against this practice (see e.g., Carranza et al. 2003; Carranza 2010). In spite of these actions, our results show a clear phylogeographic pattern in current red deer populations in Spain. Thus, our results taken together recommend preventing any restocking within Iberia, not only with non‐Iberian red deer from other subspecies, but also with specimens from the alternative Iberian lineage if we want to maintain the diversity among populations and the patterns of natural gene flow between both matrilines.

## Conflict of Interest

None declared.
